# Ultrasonic and LIDAR Sensors for Electronic Canopy Characterization in Vineyards: Advances to Improve Pesticide Application Methods

**DOI:** 10.3390/s110202177

**Published:** 2011-02-15

**Authors:** Jordi Llorens, Emilio Gil, Jordi Llop, Alexandre Escolà

**Affiliations:** 1 Department of Agri Food Engineering and Biotechnology, Universitat Politècnica de Catalunya Campus del Baix Llobregat, Edifici D4, Esteve Terradas, 8, 08860 Castelldefels, Spain; E-Mails: Jordi.Llorens.Calveras@upc.edu (J.L.); Jordi.Llop-casamada@upc.edu (J.L.); 2 Department of Agro Forestry Engineering, Universitat de Lleida Avda. Rovira Roure, 191, 25198 Lleida, Spain; E-Mail: AEscola@eagrof.udl.cat

**Keywords:** ultrasonic sensor, LIDAR, vineyard, crop adapted sprayer, leaf wall area

## Abstract

Canopy characterization is a key factor to improve pesticide application methods in tree crops and vineyards. Development of quick, easy and efficient methods to determine the fundamental parameters used to characterize canopy structure is thus an important need. In this research the use of ultrasonic and LIDAR sensors have been compared with the traditional manual and destructive canopy measurement procedure. For both methods the values of key parameters such as crop height, crop width, crop volume or leaf area have been compared. Obtained results indicate that an ultrasonic sensor is an appropriate tool to determine the average canopy characteristics, while a LIDAR sensor provides more accuracy and detailed information about the canopy. Good correlations have been obtained between crop volume (*C_VU_*) values measured with ultrasonic sensors and leaf area index, *LAI* (R^2^ = 0.51). A good correlation has also been obtained between the canopy volume measured with ultrasonic and LIDAR sensors (R^2^ = 0.52). Laser measurements of crop height (*C_HL_*) allow one to accurately predict the canopy volume. The proposed new technologies seems very appropriate as complementary tools to improve the efficiency of pesticide applications, although further improvements are still needed.

## 1. Introduction

Detailed information about canopy characteristics is a need for an adequate management of tree and vineyard crops, not only regarding pesticide application, but also for water management, fertilization schemes or pruning alternatives, all of them important aspects to achieve the main objective of a high yield and safe production. Tree canopy geometric characteristics are directly related to tree growth and productivity [1], and this information has been used for different authors to predict yield [2,3], fertilizer application in citrus crops [4], water consumption [5] or biomass [6].

The crop structure of tree or vine plants varies enormously according vegetative stage, trellis system, variety and plant density, and all those changes affect the relationship between the sprayer output and the deposit obtained on the target crop [7,8]. Pesticide applications without any consideration of crop structure are in contradiction with the general principle that foliar application should result in similar deposits, independently of crop size or canopy density [9]. This objective will lead to a considerable increase of efficacy and efficiency during the process, reducing the total amount of plant protection products required, in accord with recent EU trends [10] and avoiding the most severe problems related to environmental contamination [11,12].

Canopy characteristics can be measured manually. In this case simple values of averaged crop height and crop width are easily measured and from those values, estimations of canopy volume can be obtained. This parameter has been widely used by different authors [13–15] to establish application rates, but those manual measurements assume a homogeneous crop structure over the entire field and extrapolate measurements from several points on a crop line to the whole area. Total leaf surface and leaf area index (LAI) can be also manually measured. This involves a destructive, time consuming and expensive method including the total defoliation of a sample crop area and extended laboratory measurements of every individual leaf surface. Also in this case the obtained values in selected sampling area must be extended to the whole canopy area without consideration of any “in row” variability.

Electronic measurement of canopy dimensions in tree crops is not a new concept. In [16,17] the authors discussed the use of the ultrasonic sensors to measure canopy volume in peach and apple trees and used this information to improve the pesticide application process. The measurement system was based in a three ultrasonic sensors placed at different heights and mounted on an air-blast orchard sprayer. This work was improved furthermore by the same authors [18,19] using an advanced control algorithm. The results generated pesticide savings of up to 52% in apples.

Ultrasonic sensors transmit high frequency sound waves towards an object and sense the reflected echo. The distance between the sensor and the object is then calculated by measuring the time difference between the transmission and the reception of the waves. Distance measurements by several vertically mounted sensors have been used to calculate canopy volume in fruit, citrus and vineyard crops [20–22]. However, due to the relative wide angle divergence of ultrasonic waves [23], the field of view becomes larger as the distance between the sensor and the canopy (target) increases, reducing the accuracy of the measurements and increasing the possible interference in the signal reception of two consecutive sensors. Escolà, *et al.* [20] established that for measuring apple trees with ultrasonic sensors at distances between sensor and canopy greater than 2.0 m., the minimum distance between two consecutive ultrasonic sensors placed in a vertical pole should be 0.60 m in order to avoid interferences. In spite of these drawbacks, ultrasonic sensors have become one of the most interesting new tools to improve pesticide management in fruit and vineyard cultivation in recent years. Schumann *et al*. developed a Windows^®^ based software to manage field data obtained with ultrasonic sensors measuring tree canopy height and volume in citrus groves with a high efficiency at a rate of about 13.6 trees per minute [24].

Electronic canopy characterization allows the implementation of variable application rate techniques in fruit and vineyard crops, whereby pesticide application rates are modified according to crop characteristics as detected by the ultrasonic sensors [12,20–22,25–28]. In all those cases, canopy volume was estimated by assuming an averaged crop width for every individual crop section according the height of sensor placement on the sprayer. However, this procedure limits and introduces an error in the estimation of total volume, by assuming a constant crop width for every single crop area.

Laser sensor technology has been also adapted to determine canopy characteristics in different tree crops. LIDAR technology is a remote-sensing technique based on the measurement of the time a laser pulse takes between the sensor and a target and has the advantage that the beam can be very thin and diverges very little. In the recent years LIDAR has been used for canopy characterization in fruit trees. Tumbo *et al*. used a tree-sensing laser scanner to measure citrus canopy volume and found a good estimation of canopy volumes, especially in a grove were there are significant numbers of partially defoliated trees or small replants [29]. In [30] a measurement system to estimate the foliage surface of the crop based on a ground laser scanner was proposed, leading to the conclusion that in the estimation for a complete grove the relation between the external volume of the tree and its foliage surface can be considered linear with an average relative error of less than 6%. In [1] the authors used a laser scanner to characterize the geometric characteristics of citrus trees assuming symmetrical trees. Under those conditions they found good accuracy for the obtained results. Rosell *et al*. concluded that a LIDAR system is able to measure the geometric characteristics of plants with sufficient precision for most agriculture applications [31]. More recently Balsari *et al*. designed a sprayer prototype able to automatically adapt spray and air distribution according the characteristics of the target, to the level of crop disease and to the environmental conditions [27].

Accuracy of electronic measurements has been widely evaluated and several field tests have been developed to compare electronic canopy estimations with manual measurements. The authors of [29] compared ultrasonic and laser measurements of citrus canopy volume with manual measurement methods. They concluded that laser measurements provided better prediction of canopy volume than the ultrasonic system because of the inherent higher resolution, but in any case they recommended the use of both ultrasonic or laser sensors for automatic mapping and qualification of the canopy volume of citrus trees. Wei *at al*. developed a laser scanning system to measure canopy height, width and volume in citrus trees. In citrus trees this device showed an accuracy of 96% in length measurements in three perpendicular directions [23]. Those same authors compared laser measurements and visual assessments using a canopy boundary-smoothing algorithm, obtaining a good correlation (R^2^ = 0.96) between both methods [32]. They also found very good repeatability (coefficient of variation less than 3%) in different laser measurements. Zaman *et al*. in a comparison between ultrasonic and manual measurements of canopy volume in citrus trees, obtained differences ranging from −17.3% to 28.71% [21]. The authors also evaluated the influence of foliage density on the accuracy of electronic measurements, and concluded that volume differences were higher in light than dense trees. Arnó *et al.* used a LIDAR sensor to evaluate the leaf area index in vineyards, and results were compared with manual measurements [33]. They found a good correlation between both values, which allowed the creation of canopy maps for subsequent applications. In [34] the error in tree canopy measurements in citrus trees measured with ultrasonic sensors and a DGPS receiver was analyzed and quantified. The authors found that the most important factors affecting accuracy of the measures were DGPS ground speed, air temperature, ultrasonic performance and deviations in driving path. LIDAR and ultrasonic sensors were used also in [20] for canopy characterization on apple and pear trees. In all cases manual measurements were significantly different than those obtained with electronic devices.

The overall goal of this study was to evaluate the applicability of ultrasonic and LIDAR sensors for mapping canopy structure in different varieties and crop stages in vineyards. The specific objective was to correlate measurements of canopy characteristics using manual methods, LIDAR and ultrasonic sensors.

## 2. Materials and Methods

### 2.1. Experimental Fields

In years 2008, 2009 and 2010 several wine grape varieties, namely Cabernet Sauvignon, Tempranillo and Merlot were selected for canopy characterization at crop stages 65, 75 and 85 according to the BBCH classification [35]. Row distances ranged from 2.9 m for Merlot to 3.3 m in Cabernet Sauvignon vineyards. Field tests were arranged in two representative Spanish wine producing areas, Penedès (Barcelona) and Costers del Segre (Lleida). A total of twelve field tests were arranged from June to August every year.

### 2.2. Design of Prototype

**Figure 1.**
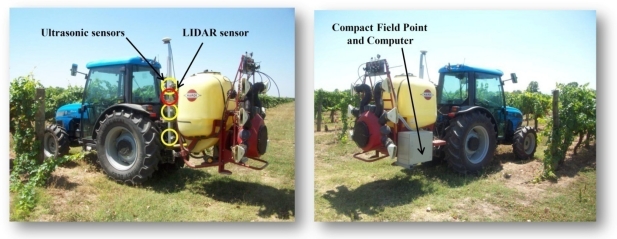
Sprayer equipped with ultrasonic sensors and LIDAR (left). The system includes a control unit with compact field point and computer to data processing (right).

Three ultrasonic Sonar-Bero sensors (Siemens AG, Munich, Germany) were placed with an equidistant spacing of 0.4 m on a stainless steel mast mounted on the left side of an air-blast orchard sprayer (Hardi LE-600 BK/2 with a centrifugal fan of 400 mm diameter). The sprayer was equipped with six individual and adjustable spouts (three on each side of the machine). The three sensors were connected to the central control unit placed on the rear part of the sprayer on which a computer and a Compact Field Point (National Instruments Corporation, Austin, TX, USA) were fitted. Data processing was done using new developed software based on LabVIEW (National Instruments) (Figure 1).

**Figure 2.**
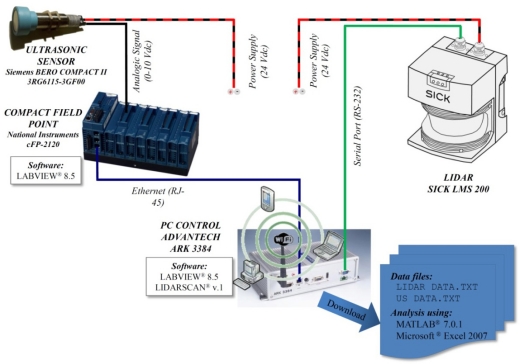
Scheme of electronic connections between all the elements installed in the prototype.

A laser scanner was also placed on the same stain-steel mast at a distance ranging from 1.40 m to 1.60 m above the ground level, depending on the canopy height. The LIDAR used was a LMS-200 model (Sick, Düsseldorf, Germany), a fully-automatic divergent laser scanner based on the measurement of time-of-flight (TOF) with an accuracy of ±15 mm in a single shot measurement and 5 mm standard deviation in a range up to 8 m [36]. The time between the transmission and the reception of the pulsed near-infrared laser beam is used to measure the distance between the scanner and the reflecting object surface. The laser beam is deflected by a rotating mirror turning at 4,500 rpm (75 rps), which results in a fan shaped scan pattern where the maximum scanning angle is 180°. The angular resolution is selectable at l°, 0.5°, or 0.25° making 181, 361 and 400 measures respectively at full scanning range with a response time of 13, 26 and 53 ms respectively. The LMS-200 has a standard RS232 serial port for data transfer with a rate selectable at 9.6, 19.2 or 38.4 Kbaud and a non-standard RS422 serial port capable of 500 Kbaud using a specific RS422 card. Selected configuration during the field tests was: angular resolution 1°, serial port RS232 and data transfer 38.4 Kbaud (Figure 2).

### 2.3. Manual Canopy Measurements

Three different canopy parameters were manually measured in each field test: crop height, crop width and leaf area index. Measuring procedure was arranged according to [37], where the total canopy height was divided into three parts. For each one of those parts height and canopy width values were obtained. Measurements were repeated 10 times on randomized vines in the whole test area. For the leaf area index calculation, all the leaves in 1 m row length (five replicates for every variety and crop stage) were picked-up separately according to the three previous levels on the canopy. Partial leaf area corresponding to each one of the three height levels was determined by applying the weight-area ratio obtained for every variety and crop stage. This ratio was determined by measuring the weight and surface area of 50 leaf samples collected from the bottom, middle and upper part of the canopy in a randomized procedure, following the method described in [28] and [38]. Leaf surface (one side) was measured with a LI-COR LI 3100C electronic planimeter.

### 2.4. Canopy Characterization with Ultrasonic Sensors

**Figure 3.**
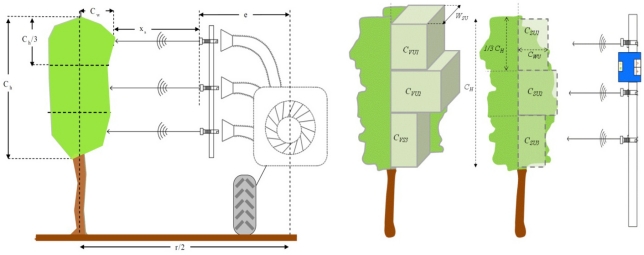
Functioning principle of ultrasonic sensors. Distance to the external layout of the crop (left) can be transformed into crop volume (right).

Ultrasonic sensors measure the distance to the external surface of the canopy by counting the lapse time between emission and reception of the emitted signal. The frequency of the pulses from the ultrasonic sensors was 20 Hz and divergence angle was 5°. The sensing range, according to manufacturer, was 400–3,000 mm and the accuracy 1.5%. Calibration curves (*x_s_* = 14.215 *v* + 181.21; R^2^ = 0.9997) was established for all three sensors, in order to verify the relation between output signal emitted, *v* (ranged from 0 to 10 V) and distance *x_s_* (m) to the external layout of the canopy. This distance was then transformed (Figure 3) into crop width (m) according Equation (1):

(1) $$C_{\mathrm{WU}}=\frac{r}{2}-e-x_{s}$$

where *C_WU_*: crop width of the semi canopy (m); *e* distance from the center of the row to the sensor (m); and *x_s_* measured distance from sensor to external layout of the crop (m).

Total and partial canopy surface for every single ultrasonic measurement was calculated according to Equation (2) in which the average canopy width measured for every ultrasonic sensor is assumed constant in all the assigned canopy height:

(2) $$C_{\mathrm{SU}}=\sum_{i=1}^{3}{\left(C_{\mathrm{WU}}\right)}_{i}\times \frac{1}{3}\times C_{\mathrm{HM}}$$

where *C_SU_* is the crop surface (m^2^); *C_WU_* the measured crop width (m) obtained from every individual sensor; and *C_HM_* the total canopy height manually measured (m).

Field measurements were carried by circulating with the tractor placed in the center of the row and driving at a constant forward speed of 1.25 m·s^−1^ (4.5 km·h^−1^). This value, together with the signal frequency of the sensor implies an average of 0.1 m of crop slice width (*W_SU_*) in the row direction for every single measurement with sensors. Multiplying this value by the estimated surface (*C_SU_*) allows to calculate the canopy volume (*C_VU_*) according Equation (3) (Figure 3):

(3) $$C_{\mathrm{VU}}=\sum_{i=1}^{L\times W_{\mathrm{SU}}^{-1}}{\left(C_{\mathrm{SU}}\right)}_{i}\times W_{\mathrm{SU}}$$

where *L* is the total length of a single crop row (m) and *W_SU_* the crop slice width for every single measurement of ultrasonic sensors (m)

In every field test one complete vine row (between 50 and 300 m length depending of the grape variety) was measured with the ultrasonic sensors by circulating in front of the both sides of the crop with the tractor moving at a constant forward speed. Using reference point measurements, total crop width (both sides) was obtained after a precise adjustment of left and right measures. Depending on measured row length the total stored values of crop width ranged from 500 to 3,000 measurements.

### 2.5. Canopy Characterization with LIDAR Sensor

**Figure 4.**
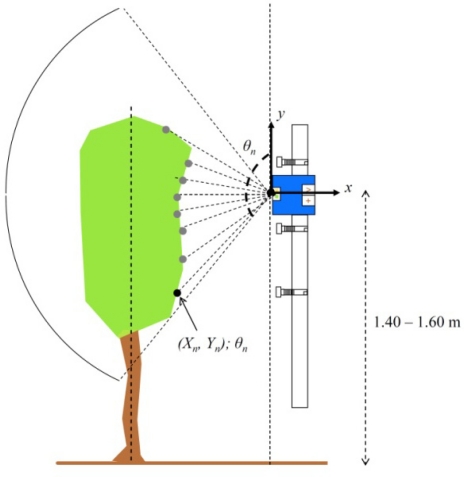
Functioning principle of the LIDAR sensor. The laser beams obtain for each crop slice a variable number of identified points according the distance to the sensor and angle from the horizontal.

**Figure 5.**
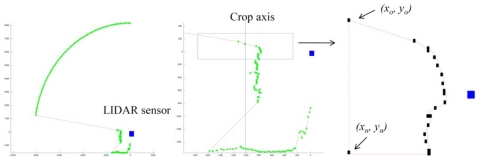
From impact points measured on the crop canopy (left) the average distance to the crop axis is calculated (middle) as crop width. Crop area is also determined for every individual height (right) from the Cartesian coordinates of every single point.

The same driving tracks along the row crop at a constant forward speed used for ultrasonic sensor measurements were used for LIDAR canopy characterization (Figure 4). Data was also stored in the on board computer in the form of polar coordinates (each point of the crop canopy was characterized by the distance and angle referred to 0° of the position of the laser sensor). Data processing was performed using *LidarScann* v.1^®^, a specific software created for management data for the SICK LSM 200 laser sensor. Furthermore, a graphic user interface was developed in MatLab (The Mathworks Inc, Natick, MA, USA) for off-line processing and algorithm development. Figure 5 shows the procedure applied to data obtained with the LIDAR sensor to determine canopy characteristics. Crop profile was first generated with all the points where the laser intercepted any leaf layout (left). Once the crop profile was obtained (centre) the average crop width measured with LIDAR, *C_WL_* (m) was calculated by the average distance of every single point to the center of the crop line. Crop height, *C_HL_* (m) was also measured with LIDAR by calculating the difference between *Y_i_* coordinates from maximum and minimum measured points on every measured range.

Once the crop profile of a single LIDAR measurement was obtained, the surface area on the perpendicular plane regarding tractor displacement was calculated applying the surface coordinates method (right) described by [39] following Equation (4):

(4) $$C_{\mathrm{SL}}=\frac{1}{2}\cdot \left[x_{0}\cdot \left(y_{n}-y_{1}\right)+x_{1}\cdot \left(y_{0}-y_{2}\right)+x_{2}\cdot \left(y_{1}-y_{3}\right)+\ldots +x_{n}\cdot \left(y_{n}-y_{0}\right)\right]$$

where *C_SL_* is the surface area on the perpendicular axis (m^2^), (*x_0_*, *y_0_*) is the Cartesian coordinates of the upper point on the profile and (*x_n_*, *y_n_*) Cartesian coordinates of the lower point of the profile

From tractor forward speed and signal frequency of the laser (100 ms^−1^) the width of every single unit of crop slice (*W_SL_*) was 0.11 m. Then, the total crop volume (*C_VL_*) was calculated integrating the volume of a single slice according Equation (5) where all slices on the total row length were included:

(5) $$C_{\mathrm{VL}}=\sum_{i=1}^{L\times W_{\mathrm{SL}}^{-1}}{\left(C_{\mathrm{SL}}\right)}_{i}\times W_{\mathrm{SL}}$$

where *W_SL_* is the crop slice width (m) with LIDAR and *L* the total length of measured row (m).

Values of crop height measured with LIDAR (*C_HL_*) were also used to determine the total leaf wall area (*LWA_L_*) on a single row (one side). This area was calculated according Equation (6):

(6) $${\mathrm{LWA}}_{L}=\sum_{i=1}^{n}{\left(C_{\mathrm{HL}}\right)}_{i}\times W_{\mathrm{SL}}$$

where *C_HL_* is the crop height (m) measured with LIDAR and *n* number of crop slices on a single crop row.

**Figure 6.**
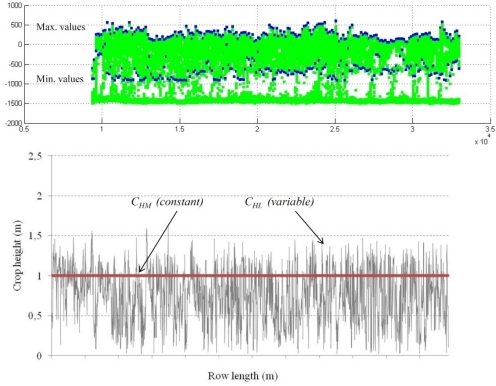
Simulated crop profile (top) obtained with laser sensor. Differences between max and min height on a single crop slice are used to measure canopy height. LWA is calculated according crop height variation along the row and compared with manual measurements (bottom).

Values of *LWA_L_* for every row were calculated according the procedure explained in Figure 6. From every LIDAR measurement on a single crop slice maximum and minimum crop height was detected and used to calculate the crop height for a single slice. According to that, the total area under the curve represents the leaf wall area of a single row (one side). Those values were compared with the similar values manually measured (*LWA_M_*).

As for statistical treatment of the data, Lillefors tests (“*Nortest*” package [40] using the statistical software R^®^ [41] have been applied for all the variables obtained with the ultrasonic and LIDAR sensors in order to check its normal distribution. In the case of non-normal distributions a Box-Cox test (“*Mass*” package) [42] from the same statistical software was applied. In those cases the value of the λ parameter was calculated and Equation (7) was applied for variable normalization prior the statistical analysis:

(7) $$X_{i}=\frac{X^{\lambda }-1}{\lambda \times {\left(X\right)}^{\lambda -1}}$$

where *X_i_* is the value of normalized variable; *X* the actual value of variable and λ the normalization constant. Correlations between different variables have been made using Pearson's product moment correlation coefficient with the “*Using R*” module [43] from the R^®^ statistical package).

## 3. Results and Discussion

### 3.1. Relationships among the Obtained Variables

The obtained average values for the most important parameters used to define the canopy structure, such as crop height, crop width and crop volume obtained with the three analyzed methods (manual, ultrasonic and LIDAR sensors) are shown in Table 1. A preliminary evaluation indicates relatively close values of canopy height measured manually (*C_HM_*) or with the LIDAR sensor (*C_HL_*). Values of crop width measured manually (*C_WM_*) were in all cases greater that those obtained with the ultrasonic sensor (*C_WU_*). Crop width obtained with the LIDAR sensor (*C_WL_*) results in the lowest values, probably due to it being the most precise scanning method and its greater ability to detect gaps in the canopy. Then, as a consequence of the observed tendency of those parameters, the measurements and estimations of crop volume present the same ranking, going from the highest values with manual determinations (*C_VM_*) to the lowest obtained with LIDAR sensor (*C_VL_*).

**Figure 7.**
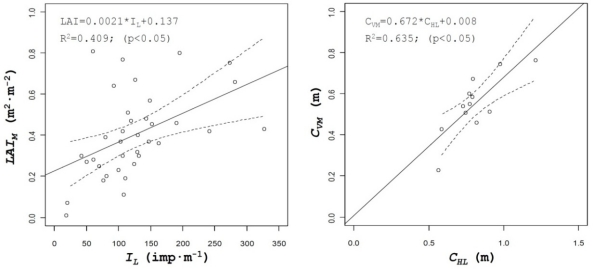
Relation between laser impact obtained with LIDAR and LAI (left). On the right correlation between canopy height calculated with LIDAR and canopy volume manually measured.

Table 2 lists the coefficients of determination of all the parameters evaluated. Those parameters included not only the above described and most important canopy structure characteristics but others, either obtained from manual measurements on the field such as leaf area index (*LAI*) or numerical values derived from the use of the sensors, such as percent of zero values measured on the crop with ultrasonic or LIDAR sensors, *Z_U_* and *Z_L_*, respectively, or *I_L_* (impacts·m^−1^) defined as the number of impacts (points were laser beam detected canopy). A good example of those relationships is shown on Figure 7, where important crop characteristics such as leaf area index of crop volume can be predicted from the number of impacts obtained with LIDAR in the canopy or from the values of canopy height measured with the same sensor, respectively.

A detailed analysis of the correlation coefficients (Table 2) of all these parameters gives precise information about which variables can be predicted by others, always with the main goal of an easy and quick canopy characterization. A selection of those relationships was made according the following criteria: (a) interesting relationships between canopy parameters and values measured electronically; (b) rational relationship between variables; and (c) variables whose coefficients of determination indicate an expected good tendency.

### 3.2. Estimation of Canopy Parameters Using Electronic Devices

**Figure 8.**
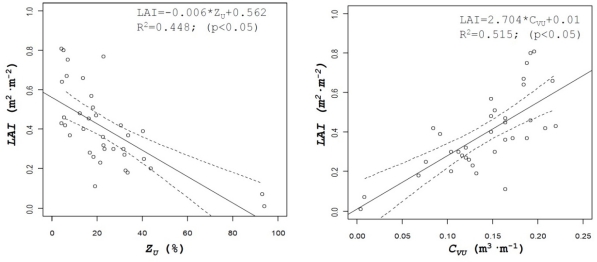

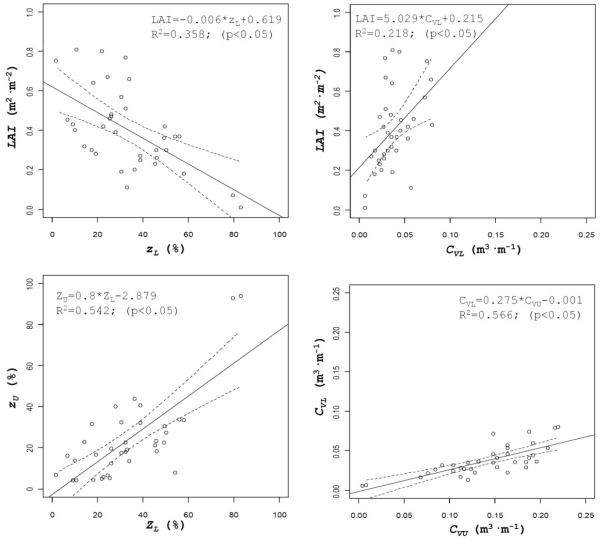
Correlation curves between measured leaf area index (LAI) and percent of zero values obtained with the two sensors (left). On the right side correlation between LAI and crop volume estimated with ultrasonic sensors and LIDAR.

Leaf area is one of the most interesting parameters used to characterize crop canopy in order to determine the most adequate volumetric rate in pesticide applications, but its determination requires in most cases destructive and time consuming methods. The use of ultrasonic and LIDAR sensors can be evaluated for the estimation of this parameter. In this research results obtained for canopy volume, either with ultrasonic sensors (*C_VU_*) or LIDAR sensor (*C_VL_*) have been compared with manual data of leaf area for all the field tests. Figure 8 shows the relationship between those parameters, giving good results for the ultrasonic sensor values (R^2^ = 0.51), being this value higher than the one obtained with LIDAR (R^2^ = 0.21).

This fact can be explained by the higher accuracy of LIDAR measurements compared to the ultrasonic sensor. For any single crop slice, canopy volume is generated with three single measurements obtained with the ultrasonic sensors, while for the same slice, LIDAR uses 180 measured points. Then there is a high probability of finding holes (gaps) into the canopy, with the consequent decrease of the calculated canopy volume. Those differences can be observed in the relationship between calculated volumes with the two sensors (Figure 8 right), where in spite of a good correlation between values (R^2^ = 0.56), differences in the measured volumes can be observed.

### 3.3. Leaf Wall Area Estimation

Leaf Wall Area (*LWA*) is one of the proposed parameters to be used during pesticide application in fruit crops [9,44,45]. Crop height values obtained with a LIDAR sensor (*C_HL_*) allow one to calculate the total leaf wall area in one side of a row canopy. The comparison of manually estimated leaf wall area (*LWA_M_*) with the values measured with the LIDAR (*LWA_L_*) is shown in Table 3. Results show that in most cases manual estimation of this parameter exceed those obtained with LIDAR by about 30%, except in some particular cases. Those differences can be related to the total row length, *L*, with average values of 0.29 m^2^·m^−1^. Those differences can affect substantially in the calculation of total amount of pesticide applied on an intended area, leading to unnecessary overdose.

## 4. Conclusions

A laser-based measurement system and three ultrasonic sensors were proposed as tools to characterize canopy structure in vineyard plantations. After three years of field tests the following conclusions can be drawn:

- The use of ultrasonic sensors allows one to obtain interesting information about crop width and its variability along the row, but limitations appear due to the range of sensor actuation and the increase of wave amplitude depending on the position. Point crop width measurements must be extrapolated to a defined canopy area, with some risk of errors.
- In spite of the difficulties mentioned above, interesting information such as canopy volume or even leaf area index can be predicted with good accuracy.
- LIDAR canopy characterization seems a very precise method. Valuable information such as percent of gaps in the canopy, variability of crop height along the row or even leaf wall area can be obtained with good accuracy. But the most difficult part of LIDAR sensor occurs during the post processing data analysis. Specific software must be developed to obtain accurate information.
- Data obtained with ultrasonic sensors is in general less precise than that obtained with LIDAR, but this fact can be compensated by the more user-friendly and easy use of US, in comparison with the sophisticated data management needed for data acquired with LIDAR sensors.

In general the field use of ultrasonic and laser sensors, together with a adequate software, seem interesting tools to improve the pesticide application process, by using all the detailed information of canopy structure in the definition of the optimal pesticide doses.

## Figures and Tables

**Table 1.** Values of most interesting crop parameters obtained with the different measurement systems.

**Wine variety**	**Crop stage^1^ (year)**	**LAI^2^**	**Crop height (m)**		**Crop width (m)**			**Crop volume (m^3^·m^−1^)**		

			*C_HM_*	*C_HL_*	*C_WM_*	*C_WU_*	*C_WL_*	*C_VM_*	*C_VU_*	*C_VL_*
***Cabernet Sauvignon***	65 (2008)	0.6	0.69	0.58	0.62	0.37	0.25	0.42	0.23	0.06
	75 (2008)	1.1	1.03	0.79	0.56	0.49	0.31	0.58	0.55	0.17
	85 (2008)	1.0	0.77	0.82	0.59	0.46	0.25	0.45	0.49	0.11
	75 (2009)	1.1	1.00	0.72	0.54	0.46	0.31	0.54	0.48	0.16
	65 (2010)	1.1	1.06	0.74	0.48	0.39	0.21	0.50	0.35	0.10
	75 (2010)	1.3	0.97	1.21	0.78	0.52	0.27	0.76	0.52	0.17
***Tempranillo***	65 (2008)	0.6	0.37	0.56	0.62	0.29	0.19	0.22	0.15	0.04
	75 (2008)	1.2	1.14	0.79	0.59	0.42	0.21	0.67	0.43	0.06
	85 (2008	1.6	0.90	0.90	0.57	0.37	0.22	0.51	0.33	0.08
***Merlot***	75 (2009)	1.7	0.94	0.76	0.64	0.46	0.12	0.60	0.50	0.09
	65 (2010)	1.8	1.06	0.77	0.52	0.45	0.30	0.55	0.48	0.08
	75 (2010)	1.5	1.02	0.97	0.73	0.46	0.29	0.74	0.48	0.15

1 According to [35]

**Table 2.** Coefficients of determination (R^2^) among crop parameter values obtained with the three measurement systems.

			**Manual**		**Ultrasonic sensor**					**LIDAR sensor**			
		C_HM_	C_WM_	LAI	C_VM_	C_WU_	C_VU_	Z_U_	C_HL_	C_WL_	C_VL_	Z_L_	I_L_
**Manual**	C_HM_	1											
	C_WM_	0.02	1										
	LAI	0.41**^2^**	0.08	1									
	C_VM_	0.66	0.30	0.23	1								
**Ultras.**	C_WU_	0.55	0.04	0.42	0.09	1							
	C_VU_	0.55	0.04	0.42**^1^**	0.09	1	1						
	Z_U_	0.66	0.00	0.44**^1^**	0.13	0.77	0.77	1					
**LIDAR sensor**	C_HL_	0.23	0.37	0.29	0.64**^1^**^,^**^3^**	0.44	0.37	0.33	1				
	C_WL_	0.09	0.00	0.00	0.00	0.05	0.05	0.00	0.06	1			
	C_VL_	0.21	0.01	0.22**^1^**	0.01	0.57	0.57**^2^**	0.38	0.37	0.16	1		
	Z_L_	0.26	0.00	0.36**^1^**	0.07	0.41	0.41	0.54**^2^**	0.08	0.02	0.26	1	
	I_L_	0.26	0.08	0.40**^1^**	0.17	0.55	0.55	0.38	0.43	0.03	0.77	0.21	1

Selection criteria:

1 interesting relationship;

2 rational relationships;

3 good correlations is expected

**Table 3.** Values of leaf wall area (LWA) for the whole row length (one side) manually measured and electronically estimated with LIDAR. Relative differences (%) and by row length unit (m).

**Wine variety**	**Row length *L* (m)**	**Leaf Wall Area *LWA* (m^2^)**			***(LWA_M_–LWA_L_)/ L***
		*LWA_M_*	*LWA_L_*	(LWA_M_ − LWA_L_)*100/ LWA_M_	
***Cabernet Sauvignon***	124.4	85.8	42.5	50.5%	0.348
	126.8	131.0	100.0	23.7%	0.245
	118.7	91.4	71.9	21.3%	0.164
	330.0	330.0	245.6	25.6%	0.256
	331.2	351.1	232.5	33.8%	0.358
	338.7	330.2	421.8	−27.7%	−0.270
***Tempranillo***	50.0	18.5	26.5	−43.0%	−0.159
	52.8	60.2	28.5	52.6%	0.600
	47.8	43.0	30.0	30.4%	0.273
***Merlot***	205.0	192.7	176.2	8.6%	0.081
	205.7	218.0	155.5	28.7%	0.304
	204.3	208.3	236.8	−13.6%	−0.139
